# dbMPIKT: a database of kinetic and thermodynamic mutant protein interactions

**DOI:** 10.1186/s12859-018-2493-7

**Published:** 2018-11-27

**Authors:** Quanya Liu, Peng Chen, Bing Wang, Jun Zhang, Jinyan Li

**Affiliations:** 1 Institute of Physical Science and Information Technology, Anhui University, Hefei, 230601 Anhui China; 2 School of Electrical and Information Engineering, Anhui University of Technology, Ma’anshan, 243032 Anhui China; 3 School of Electronic Engineering & Automation, Anhui University, Hefei, 230601 Anhui China; 4 Advanced Analytics Institute and Centre for Health Technologies, University of Technology, Broadway, Sydney, NSW 2007 Australia

**Keywords:** Protein-protein interactions, Mutants, Kinetic data, Thermodynamic data

## Abstract

**Background:**

Protein-protein interactions (PPIs) play important roles in biological functions. Studies of the effects of mutants on protein interactions can provide further understanding of PPIs. Currently, many databases collect experimental mutants to assess protein interactions, but most of these databases are old and have not been updated for several years.

**Results:**

To address this issue, we manually curated a kinetic and thermodynamic database of mutant protein interactions (dbMPIKT) that is freely accessible at our website. This database contains 5291 mutants in protein interactions collected from previous databases and the literature published within the last three years. Furthermore, some data analysis, such as mutation number, mutation type, protein pair source and network map construction, can be performed online.

**Conclusion:**

Our work can promote the study on PPIs, and novel information can be mined from the new database. Our database is available in http://DeepLearner.ahu.edu.cn/web/dbMPIKT/ for use by all, including both academics and non-academics.

**Electronic supplementary material:**

The online version of this article (10.1186/s12859-018-2493-7) contains supplementary material, which is available to authorized users.

## Background

Protein-protein interactions (PPIs) play crucial roles in organisms particularly by mediating the majority of biological functions [1]. Mutations in PPIs are associated with some human diseases, for instance, cancer and Alzheimers disease [2]. In some studies, the mechanism of PPIs has been investigated and used for treat intervention and drug design [3, 4]. PPI interfaces contain many amino acid residues, but only a few of these amino acids greatly contribute to binding free energy, which are defined as hot spots [5]. Hot spots can be determined by the calculation of mutant data on protein interactions. The knowledge of hot spots is extremely important in designing PPI inhibitors [6]. Many researchers have developed different methods to obtain mutant information on protein-protein interactions and have built public databases for users to investigate hot spots [7].

Traditionally, hot spots can be determined using biological experiments, such as alanine scanning mutagenesis and alanine shaving [8]. In general, residues with alanine mutations that exhibit changes in binding free energy (G) of 2.0 kcal/mol are defined as hot spots (HS), whereas others are defined as nonhot spots (NS) [9]. Several studies have attempted to build mutation databases associated with hot spots. The first database of alanine mutations in protein interactions named ASEdb was built by Thorn and Bogan [10], and experimentally determined binding affinity data were collected. Then, BID was developed by Fischer et al. This database extracted hot spots in protein interfaces from scientific literature [11]. Kumar and Gromiha built the PINT database, which mainly stored thermodynamic data on PPIs, such as binding free energy change, dissociation constant, and heat capacity change [12]. SKEMPI is a manually curated database containing 3046 binding free energy changes upon mutation in the literature [13].

However, experimental methods for hot spot identification are time- consuming and labor-intensive. In addition, it is also difficult to measure all potential binding hot spots in a large number of proteins [14, 15]. Therefore, many researchers have developed computational tools to identify hot spots. Machine learning methods were most widely used in the related fields of hot spot identification, such as SVM, Random Projection, and Random Forest [16–21]. The group used existing databases to build a training model and further applied this model to predict potential hot spots from unknown amino acid residues [22]. In addition, these hot spot residues can be used to identify the effects of protein-protein affinity changes when missense mutations occur. Some researchers have combined sequence- and structure-based methods to judge the effect of point mutations on protein-protein affinity using the change in free energy [23]. Furthermore, some studies have attempted to study the effects of single or multiple missense mutations on protein-protein affinity. Li et al. improved predictive performance by changing energy functions or adjusting parameters [24]. However, in recent years, these databases were not maintained and updated in a timely manner. To address this issue, we built a state-of-the-art database by mining mutants of protein interactions from related databases and literature.

This work presents a kinetic and thermodynamic database of mutant protein interactions called dbMPIKT. The database consists of data from previous databases about mutant protein interactions, including BID, SKEMPI and AB-Bind, and data extracted from scientific literature published in recent years. The dbMPIKT contains 5291 nonredundant mutants of experimental kinetic and thermodynamics data upon mutation. Our database will facilitate research on hot spot prediction, drug discovery, and other topics.

## Construction and content

### Data collection

**Fig. 1:**
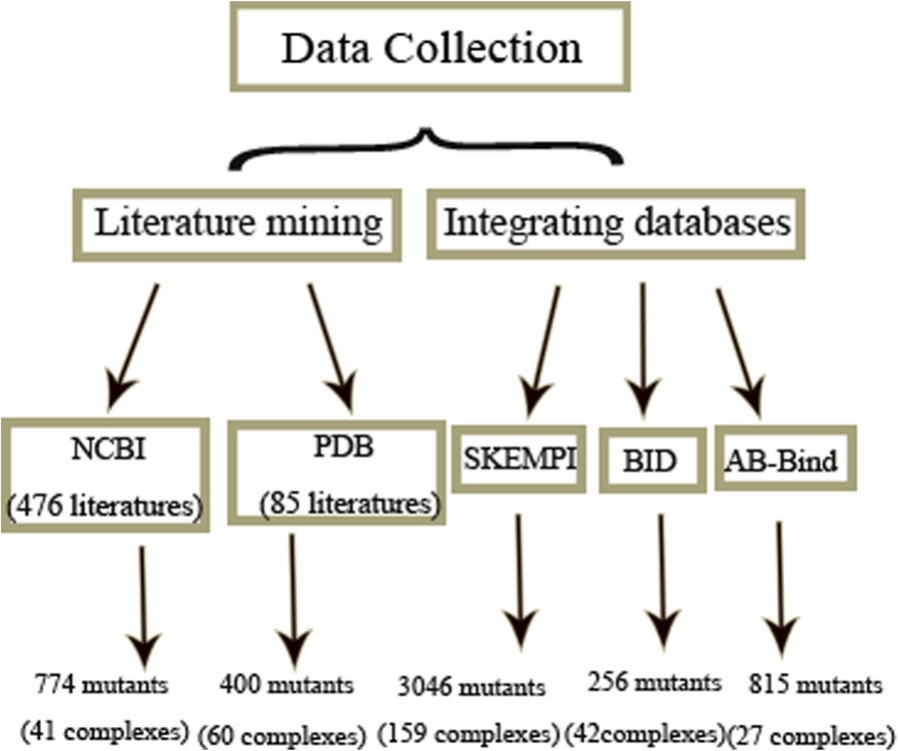
The flowchart of data collection

This database consists of two types of data sources. On data source involves existing databases, i.e., SKEMPI, BID, and AB-Bind; the other data source is curated literature. Our curated literature database collected the mutation data of protein interactions from scientific literature within the past three years (The detailed literature can be found in Additional file 1: Figure S1). To build the curated database, first, a comprehensive literature search method was performed to identify related literature in PubMed using two sets of keywords. One set contains the terms of PPIs, G and thermodynamics data, and the other set contains the terms of PPIs, amino acid mutations and kinetic data. The kinetic and thermodynamics data of mutants were curated from PubMed literature. Although some of the studies were missed, 425 credible studies were obtained. Figure 1 shows the detailed information of data collection.

Then, the structures of protein complexes were obtained by advanced searches of the PDB database using various query items, i.e., macromolecule type (only contains protein), protein stoichiometry (heterodimer complexes), release date (from1 January 2013 to 31 December 2016) and X-ray resolution (less than 3^°^A). As a result, 1017 protein structures were obtained from 682 citations in PDB, which were mapped to the PDB-Bind database to extract the corresponding thermodynamic data. A total of 99 complex structures from 85 citations containing dissociation constant (*Kd* value) information were obtained. All of the literature was manually assessed, and all Kd values of the structures were recorded [25]. The details of the collection of protein complexes and their sources can be referred to the Additional file 2: Figure S2.

After removing redundancy based on the above procedure, our database contains 5291 mutations that are composed of manually curated data and the three existing databases.

### Database construction

The dbMPIKT database is available online and is composed of some functional modules, such as query, statistics and analysis. For example, a quick search is located on the top right of the homepage. Users can search for a target protein in the database and obtain relevant mutant information using PDB ID. Additionally, users can find statistical information in the database and links to related websites in the homepage of dbMPIKT.

**Fig. 2:**
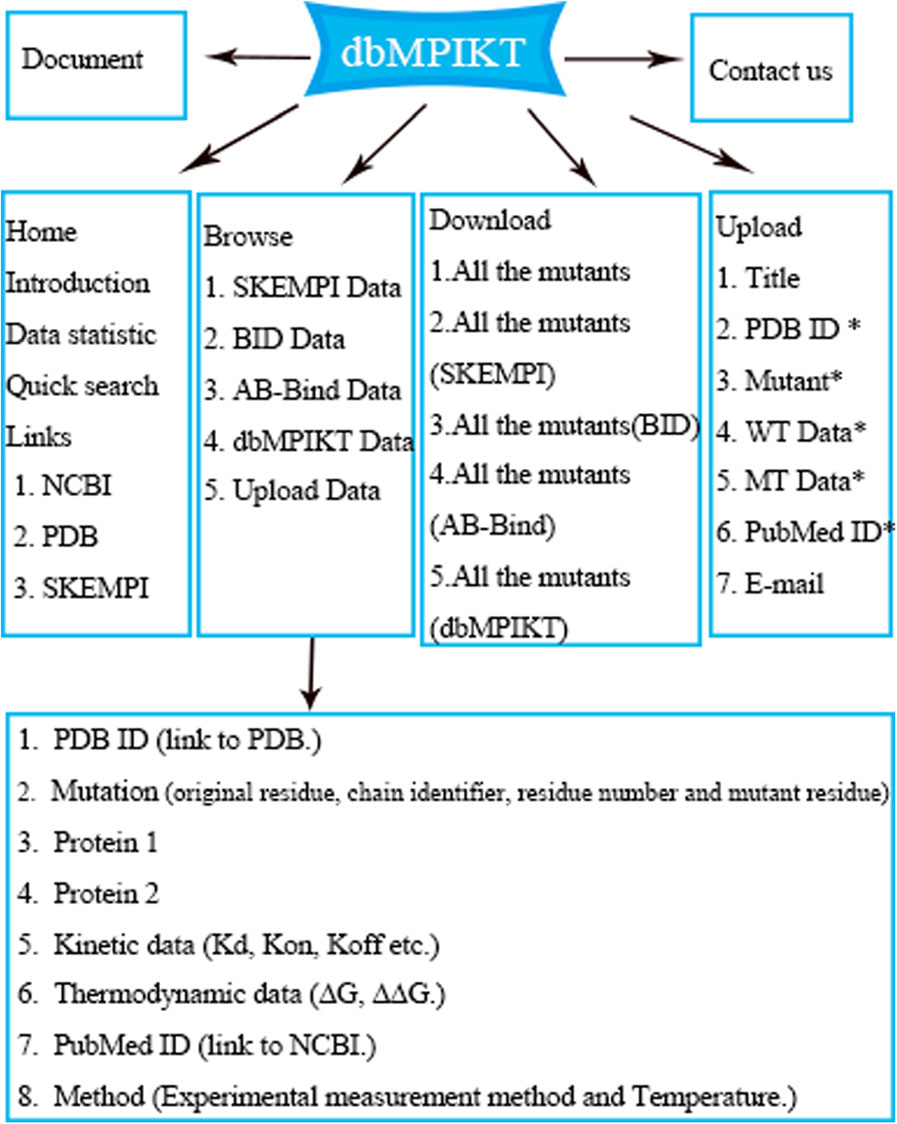
The database structure of dbMPIKT

The webserver includes the following pages: home, browse, document, upload, download and contact. Figure 2 presents the entire database structure. The Browse page presents all data in the database. Here, you can see the details of mutants from the four sources. All data can be freely downloaded. To continuously update the database, an upload link is provided to help users upload their own data that is subsequently assessed and stored in the database through a user-friendly interface. In addition, the newly uploaded data are also presented on the browse page.Our dbMPIKT was constructed using MySQL and PHP. More information about the database can be obtained by browsing the six webpages.

### Analysis of protein-protein interaction pairs and interaction network construction

In addition to mutation data collection, related protein-protein interaction pairs were also recorded in our database. All protein-protein complexes were classified into different categories based on atomic structures of complexes. In addition, to illustrate whether each pair of PPIs is linked, a network analysis tool (Cytoscopeversion 3.5.1) [26] was embedded into dbMPIKT to construct interaction networks.

According to the network map, some features of PPI network, such as the regularity of PPIs, can be obtained by analyzing the association of PPIs and network structure.

## Utility and discussion

### Important features in database

In this paper, although data entries in dbMPIKT were obtained from different sources, the database contains distinguished attributes. The first feature is the PDB ID, which denotes the ID of the protein-protein complex in the PDB database. This ID is linked to a related PDB website, so users can obtain more information on the complex. The second attribute is mutation information, which consists of original residue, chain identifier, the position of the mutant residue in sequence and the name of mutant residue. The third attribute includes the names of the two interacting proteins, namely protein 1 and protein 2. Additional attributes in- clude kinetic data and thermodynamic data. In general, kinetic data (*Kd*), includes the association rate (*Kon*), and dissociation rate (*Koff*). Most data are presented in units of *nM*, *M*^*−* 1^*S*^*−* 1^ and *S*^*−* 1^. Other units can be converted into these units. Moreover, thermodynamic data contain changes in binding free energy (∆G) and differences in binding free energy changes between the mutant and wild-type complex (∆∆G). These values are reported as kcal/mol. PubMed ID is another attribute. This ID is the source of kinetic and thermodynamic data. In addition, you can refer to more details by clicking on each PubMed ID in the table and download literature from NCBI. The last attribute is Method, which presents the experimental measurement method of the affinity of PPIs. There are mainly two methods: SPR (surface plasmon resonance) and ITC (isothermal titration calorimetric) [27]. Temperature information is also included as an attribute. The other three databases contain data attributes similar to our curated database, and users can be referred to corresponding literature.

### Database statistics

**Fig. 3:**
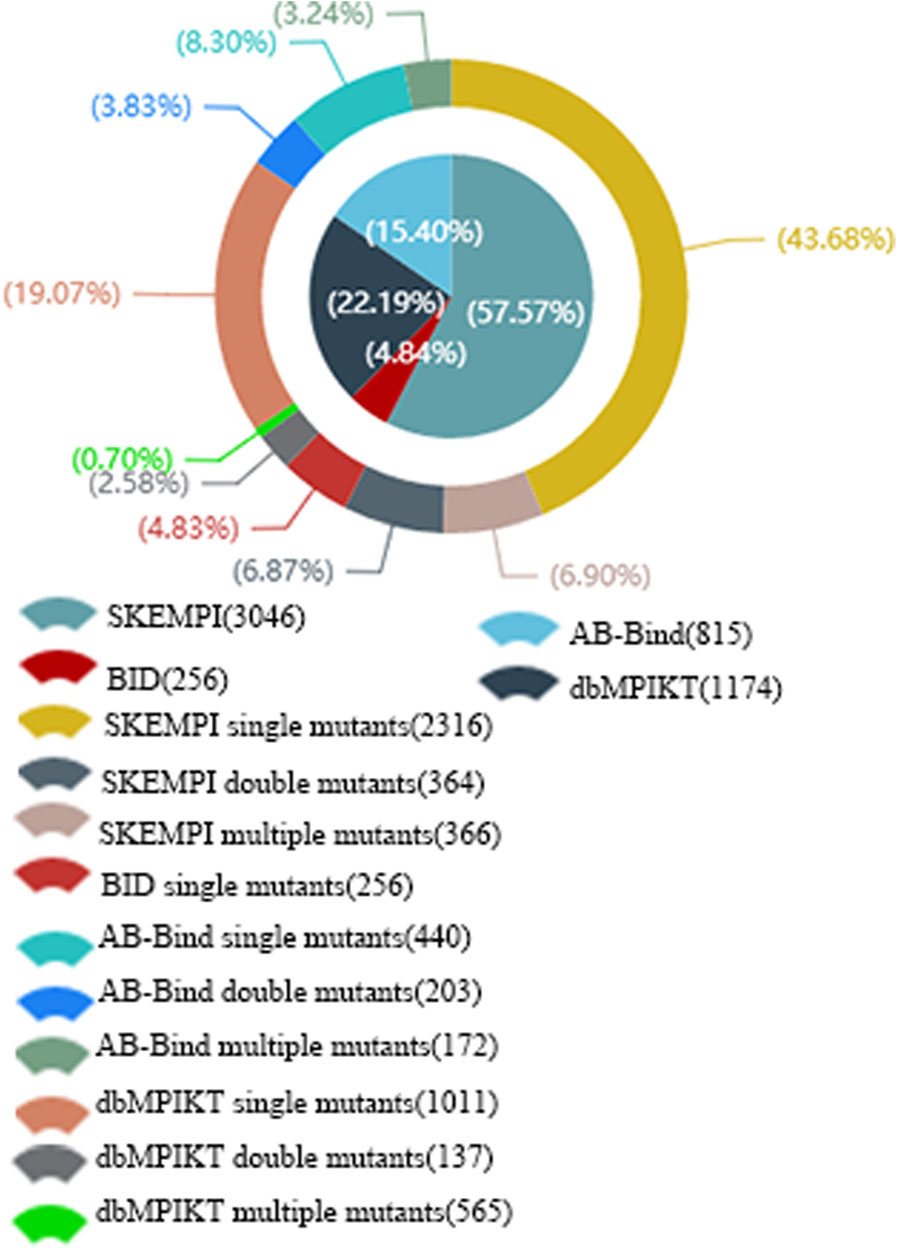
The data distribution of the four data sets

The dbMPIKT database collected 5291 mutants with kinetic or thermodynamic data. The data were divided into four sources: SKEMPI, AB-Bind, BID and literature, containing 3046, 815, 256 and 1174 mutants, respectively. The mutants are derived from 233 structures of 245 protein-protein complexes, and only 12 complexes do not have PDB IDs. Some statistical information of dbMPIKT can be found on our website, where the comparison of the four databases with respect to mutation type is presented. The mutations in each database are clustered into three mutation types: single mutants, double mutants and multiple mutants. The data distribution from different sources is presented in Fig. 3 (More details can be found in Additional file 3: Table S3 of supplementary materials). In general, the SKEMPI database contains the greatest number of single mutants, and the curated database contains the second most single mutants. Regarding mutation type, single mutations account for 75.88% of the total mutations, double mutations account for 13.28% and multiple mutations account for 10.84%.

Specifically, the collected thermodynamic and kinetic data are measured by G, change in enthalpy (H), change in entropy (S), and kinetic rate constants. These values are derived from SPR, ITC and alanine mutation scanning (AMS) experiments.

The database contains almost all experimental mutants to date. For single mutations, we counted the number of mutations for each type of amino acids. Table 1 presents the distribution of 20 types of amino acids in single mutant data (More details can be found in Additional file 4: Table S4 of supplementary materials). Statistically, alanine mutation accounts for 56% of single mutant data, and threonine has the lowest mutant rate. Compared with other data sets, these results are more commonly observed in the curated database, where alanine mutations account for 66.7% of all mutations. In terms of amino acid properties, the 20 types of amino acids are divided into five categories: polar (S, T, N and Q), hydrophobic (A, I, L, M, V, W, Y and F), positive (R, K and H), negative (D and E) or other (G, P and C) [28].

**Table 1:** The distribution of single mutant amino acids for the four datasets

Dataset	Amino acid	SKEMPI	BID	AB-Bind	dbMPIKT	Total
Hydrophobic	A	1057	256	267	673	2253
	I	53	0	2	11	66
	L	77	0	3	17	97
	M	60	0	3	12	75
	V	74	0	12	12	98
	W	5	0	14	13	81
	Y	56	0	14	14	84
	F	94	0	8	23	125
	S	71	0	11	21	103
Polar	T	48	0	6	9	63
	N	60	0	15	21	96
	Q	78	0	25	21	124
Positive	R	76	0	15	18	109
	K	98	0	7	29	134
	H	54	0	2	10	66
Negative	D	76	0	15	27	118
	E	83	0	12	35	130
Other	C	45	0	1	14	60
	G	59	0	7	21	87
	P	78	0	25	21	124
Total		2316	256	440	1010	4022

### Analysis of protein-protein pairs in dbMPIKT

**Fig. 4:**
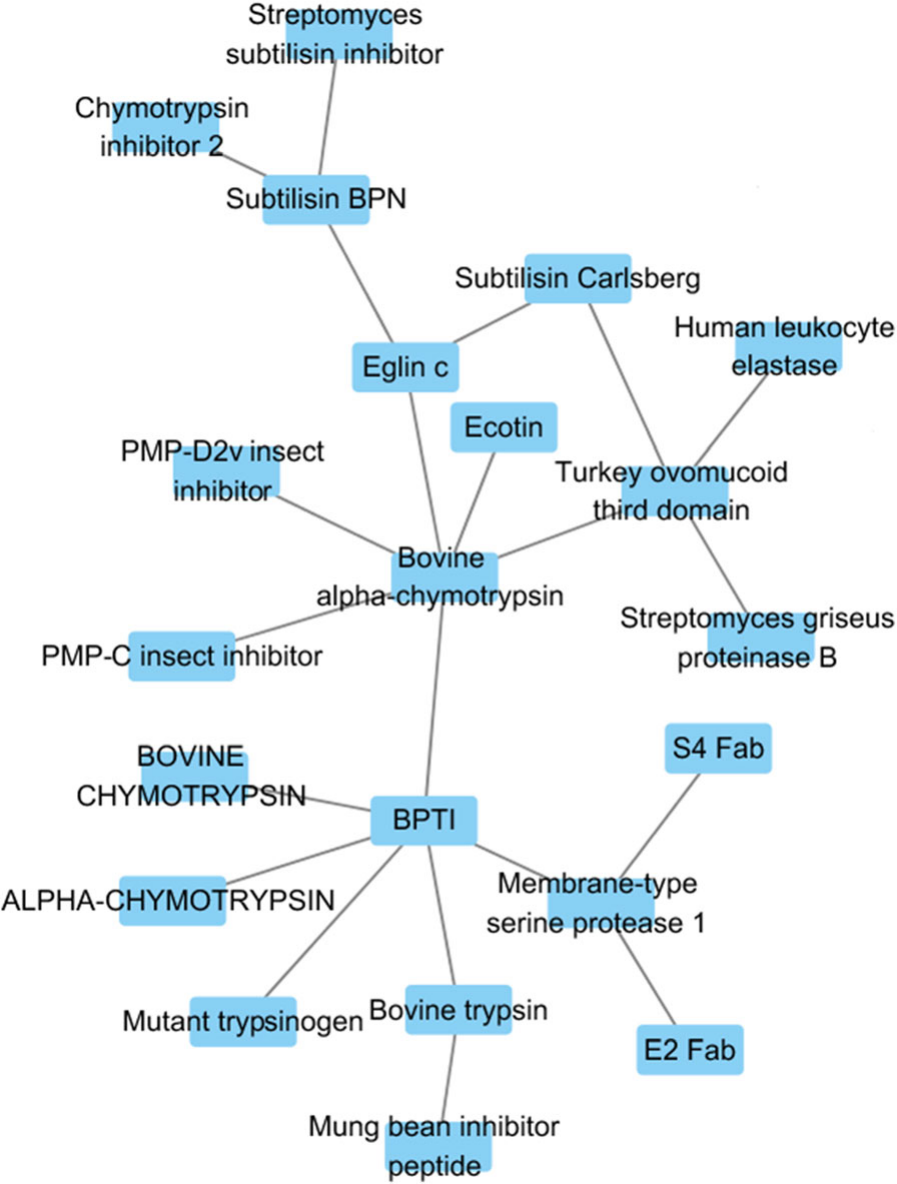
Part of interactions on the network map. Each node in the picture represents a protein, and the connection between nodes represents an interaction

In our database, 5291 mutants were obtained from 245 protein-protein complexes, including heterodimer complexes, antigen-antibodies, and enzyme-inhibitors [29]. In addition, human, *Mus musculus*, and *Bos taurus* proteins are included in the database, and human proteins represent the largest group. A protein interaction network was constructed based on protein interaction pairs, which can be used to identify protein functions for specific protein interactions [30]. Figure 4 illustrates a part of the protein interaction network, and the entire network is presented in Additional file 5: Table S5 of the supplementary material. In Fig. 4, most of the protein interactions are independent, but it is interesting that a small portion of proteins interact with each other to form an interaction network. Figure 4 demonstrates seen that a small network is centered at basic pancreatic trypsin inhibitor (BPTI) and bovine alpha-chymotrypsin protein, which are both *Bos taurus* proteins. BPTI plays an important role in biomedical science given that it can be used to study the conformations and PPIs of globular proteins reduce hemorrhagic complications in clinical practice [31]. Furthermore, the protein interaction network is an important tool to analyze the biological function of proteins [32].

### Data source analysis

The dbMPIKT consists of data from four data sources, which all include kinetic or thermodynamic data of mutant protein interactions. However, these data are somewhat different. The SKEMPI database contains the largest number of mutants, and the manually curated database is the second largest source. The BID is the least represented source given that the BID database is not currently operational and data cannot be downloaded directly. Some BID data are extracted from the additional studies in the literature [33]. In addition, our curated database contains the largest number of alanine mutations in terms of mutant types. Therefore, our database is more useful for hot spot predictions. Moreover, based on protein types, previous databases almost exclusively targeted specific complexes. For example, AB-bind is an antibody binding mutational database extracted from information regarding antigen-antibody complexes. Our work integrated these databases together so that it is easy for researchers to obtain required data. Although SKEMPI has been updated recently, i.e., SKEMPI2.0 [34], the description of mutation data in our database is more consistent with scientific research compared with SKEMPI2.0. To clearly describe the characteristics of mutation data in our curated database, mutation data features are classified into two simple categories: wild type data (WT data) and mutated data (MT data). Among them, each type of data contains thermodynamic data or kinetic data.

### Biological significance of database data

Protein-protein interactions have been extensively studied, and many researchers have proposed calculation methods for PPI predictions. Among them, disease– related PPIs deserve in-depth study [35]. Our database provides information on mutant data and PPI pairs as well as links to related websites that can indirectly capture structure and sequence information for each protein complex. This information can be used as features for PPI predictions. For example, evolutionary features can be obtained from protein sequences and incorporated into Ensemble to predict hots spots [16, 19]. Protein pairs also represent an important part of our database, i.e., self-interacting proteins (SIP) are a type of PPI, and SIP detection is a recent hot topic of related research [36]. In general, our database can provide valid datasets and relevant feature information for PPI predictions.

## Conclusions

The paper proposes to integrate the three previous databases and manually curated data presented in the literature over the last three years. We built a web server to store kinetic and thermodynamic data on mutant protein interactions. More detailed information about mutants and protein-protein interactions can be found on the web server. In our database, kinetic and thermodynamic data of mutants, including *Kd*, ∆∆G, ∆G, *Koff* and *Kon*, are obtained. In addition, some data can be calculated using other data. For example, ∆∆G, a parameter that can be used to diametrically distinguish hot spots and nonhot spots, can be indirectly obtained using the following equation:

1 $$ \mathrm{Kd}=\frac{K_{\mathrm{off}}}{K_{\mathrm{on}}} and\kern2em \Delta \mathrm{G}=\mathrm{RT}\kern0.5em \ln \kern0.5em {\mathrm{K}}_{\mathrm{d}.} $$

The database provides a large hot spot data set that can help improve the applications of hot spots and hot spot predictions.

### Webserver

Our free website is available at http://DeepLearner.ahu.edu.cn/web/dbMPIKT/. Users can perform advanced searches on the home page to obtain interesting data and browse all data on the browse page.

## Additional files

Additional file 1: **Table S1.** Literature list for the collected data. (XLSX 60 kb)

Additional file 2: **Table S2.** The collection of protein complexes and their sources. (XLSX 38 kb)

Additional file 3: **Table S3.** Distribution of mutation types. (XLSX 10 kb)

Additional file 4: **Figure S4.** Mutation distribution of amino acid types. (PNG 22 kb)

Additional file 5: **Figure S5.** Protein interaction network map. (PNG 71 kb)

## Abbreviations

AB- Bind: Antibody binding mutational database
ASEd- b: Alanine Scanning Energetics database
BID: Binding Interface Database
dbMPIKT: Kinetic and thermodynamic database of mutant protein interactions
G: Binding free energy
HS: Hot spots
ID: Identification
NS: Non-hot spots
PDB: Protein Data Bank
PINT: Proteinprotein Interactions Thermodynamic
PPIs: Protein-protein interactions
SVM: Support Vector Machine
