# Supplementary figures and images for: dbMPIKT: a database of kinetic and thermodynamic mutant protein interactions
Source: BMC Bioinformatics. 2018 Nov 27;19:455. doi: 10.1186/s12859-018-2493-7 (PMC6260753; doi:10.1186/s12859-018-2493-7)

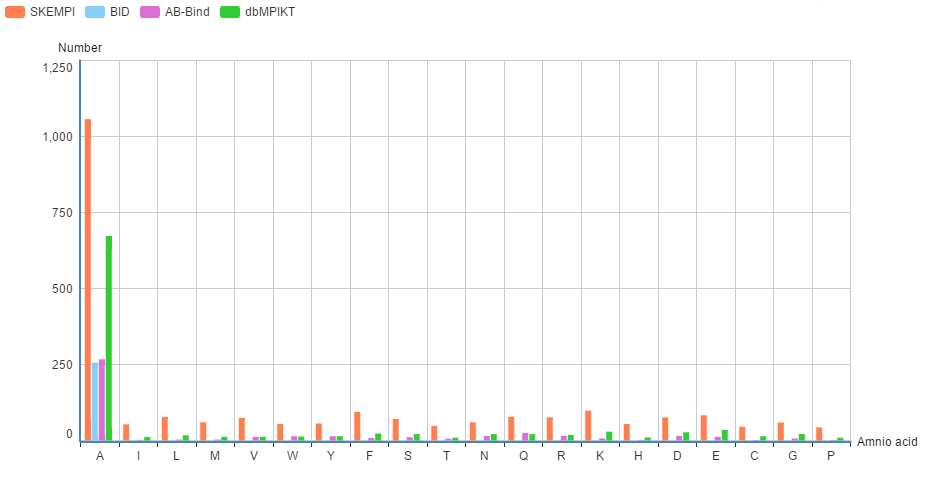

Supplement: Supplementary file 4 — Figure S4. Mutation distribution of amino acid types. (PNG 22 kb) [file 12859_2018_2493_MOESM4_ESM.png]

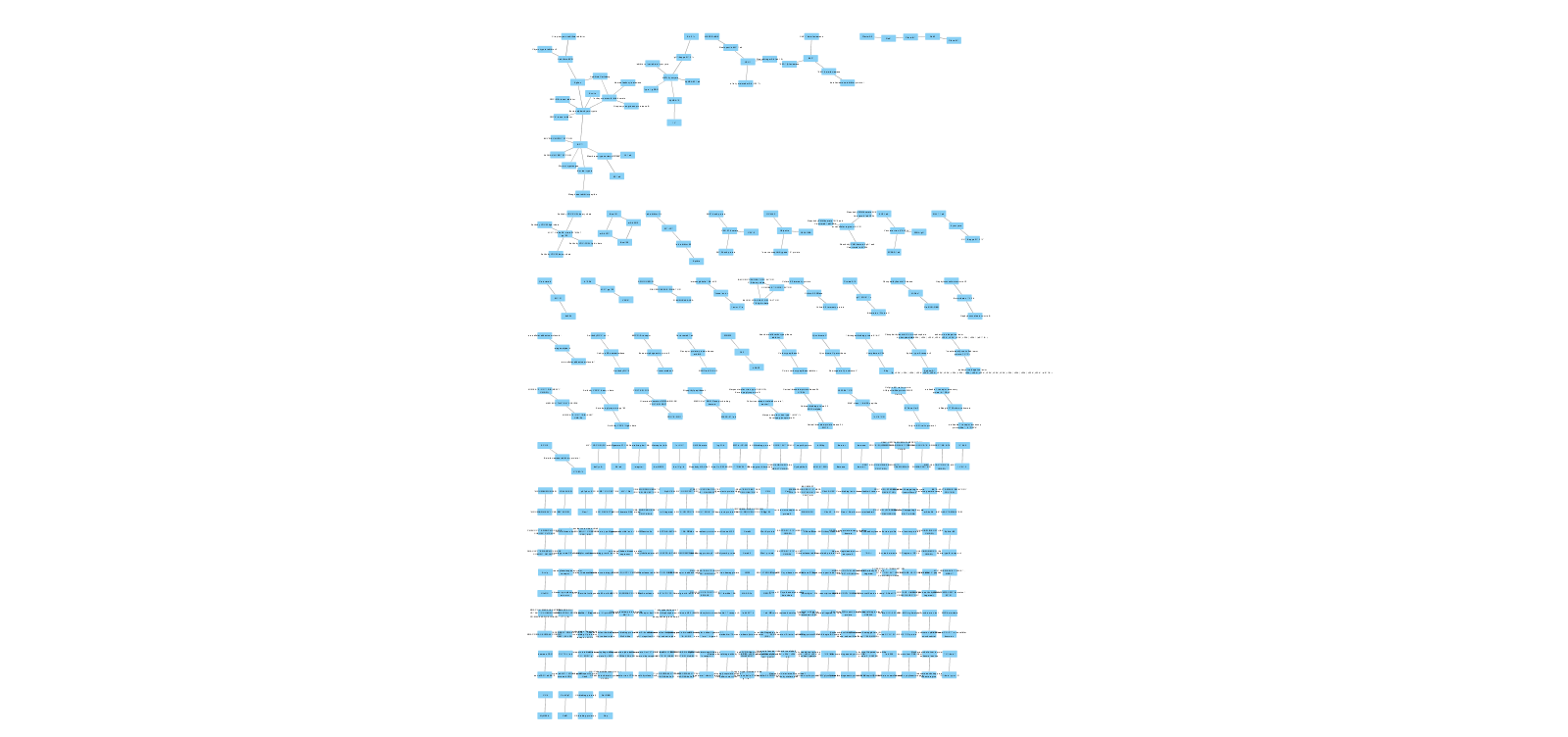

Supplement: Supplementary file 5 — Figure S5. Protein interaction network map. (PNG 71 kb) [file 12859_2018_2493_MOESM5_ESM.png]
